# Diabetic kidney disease macrophage cholesterol efflux: a revolution from metabolism to immune

**DOI:** 10.3389/fendo.2025.1714167

**Published:** 2025-11-06

**Authors:** Jinjin Wang, Yi Cai, Yuxi Feng, Qin Zhu

**Affiliations:** 1 Department of Nephrology, Hangzhou Traditional Chinese Medicine Hospital Affiliated to Zhejiang Chinese Medical University, Hangzhou, China; 2 The First School of Clinical Medicine, Zhejiang Chinese Medical University, Hangzhou, China

**Keywords:** diabetic kidney disease, macrophages, lipid metabolism disorders, M1/M2 phenotype, LXR/PPARγ signaling

## Abstract

Diabetic kidney disease (DKD) is the most common microvascular complication of diabetes and a leading cause of end-stage renal disease (ESRD). Traditionally, its pathogenesis has been attributed to hyperglycemia-induced metabolic disturbances, glomerular hyperperfusion and hyperfiltration, activation of the renin–angiotensin–aldosterone system (RAAS), and oxidative stress. Recent evidence, however, indicates that chronic inflammation and immune dysregulation also play critical roles in DKD progression.Impaired macrophage cholesterol efflux (MCE) has emerged as a central pathogenic mechanism in DKD. Under hyperglycemic conditions, advanced glycation end-products (AGEs) suppress the LXR/PPARγ signaling pathway and downregulate downstream transporters ABCA1 and ABCG1, thereby reducing cholesterol efflux. This disruption promotes lipid accumulation and macrophage foam cell formation, leading to the sustained release of pro-inflammatory cytokines such as TNF-α, IL-1β, and MCP-1, which accelerate glomerulosclerosis and tubulointerstitial fibrosis. MCE dysfunction thus provides a mechanistic link bridging metabolic dysregulation and immune-mediated inflammation in DKD.Therapeutic strategies targeting MCE show promising potential. Pharmacological agents such as LXR/RXR agonists, PPARγ activators, sodium-glucose cotransporter 2(SGLT2) inhibitors, and glucagon-like peptide-1 receptor agonists(GLP-1RAs) enhance cholesterol transport, promote macrophage polarization toward the M2 anti-inflammatory phenotype, and ameliorate renal injury. In addition, natural bioactive compounds and nanodelivery systems can selectively modulate ABCA1/G1-mediated cholesterol efflux, attenuating lipid accumulation.In conclusion, this study highlights the pivotal role of macrophage cholesterol efflux in DKD pathogenesis beyond traditional metabolic factors and proposes novel MCE-targeted therapeutic strategies, offering new insights for the prevention and treatment of DKD.

## 1 Introduction

Based on data from the IDF in 2019, it is estimated that approximately 463 million adults aged 20–79 worldwide are affected by diabetes, with projections indicating that this number will increase to 578.4 million by 2030. The global range of end-stage renal disease (ESRD) cases attributed to diabetes is between 10% and 67% (1). Diabetic kidney disease (DKD), a significant microvascular complication of diabetes, is a leading cause of ESRD. Recent studies have identified a strong correlation between the pathogenesis of DKD and inflammatory and immune responses. Macrophages, serving as the primary inflammatory cells, are integral to advancing DKD (2). The hyperglycemic milieu resultant from diabetes can prompt M1 polarization of macrophages, influence macrophage cholesterol efflux (MCE), and prompt their transformation into foam cells, thereby exacerbating DKD (3). This study delves into the issue of lipid metabolism disturbances in macrophages triggered by DKD, scrutinizes the physiological mechanisms of MCE, investigates the correlation between impaired MCE and DKD progression, and outlines potential strategies for reinstating MCE with the objective of retarding the progression of DKD.

## 2 The biological basis of macrophage cholesterol efflux

### 2.1 The mechanisms of macrophage cholesterol efflux

Macrophages are essential components in lipid metabolism within the human body, participating in various processes such as lipid uptake, storage, metabolism, and degradation. MCE is the term used to describe the mechanism by which macrophages transport cholesterol to the extracellular space, a vital process for regulating intracellular cholesterol levels and preventing lipid accumulation (4). Macrophage surface receptors, particularly scavenger receptors (SR), have the capability to internalize oxidized low-density lipoprotein (ox-LDL). The absorbed lipids are subsequently sequestered as lipid droplets within macrophages and are mobilized as necessary following enzymatic degradation by lipases and cholesterol esterases (4). Liver X receptor (LXR) and peroxisome proliferator-activated receptor gamma (PPARγ) play pivotal roles as transcription factors in the regulation of macrophage cholesterol metabolism. They enhance MCE by inducing the upregulation of ATP-binding cassette transporters A1 (ABCA1) and G1 (ABCG1) (5, 6). Activation of cholesterol-dependent LXR/retinoid X receptor (RXR) transcription factors occurs when there is a need to export lipids from macrophages or in cases of lipid overload. These transcription factors target the DR4 sites within the proximal promoters of the ABCA1 gene, thereby modulating the expression of lipid transport proteins like ABCA1 on the macrophage surface (7). The ABCA1-mediated pathway is the principal mechanism by which cholesterol is transported out of macrophages (8, 9). This pathway involves the creation of a plasma membrane microdomain by ABCA1, which aids in the transfer of phospholipids and cholesterol to apolipoprotein A-I (ApoA-I). Subsequently, the lipidated ApoA-I, also known as nascent high-density lipoprotein (HDL) particles, acquire additional cholesterol through the ABCG1-mediated efflux pathway, leading to the formation of mature HDL particles. These mature HDL particles then interact with the scavenger receptor class B type I (SR-BI) on the cell surface. The extracellular domain of SR-BI functions as a nonpolar channel facilitating cholesterol exchange (10–12). Studies have shown that SR-BI exhibits the greatest binding affinity for large, spherical HDL particles. In contrast, ABCA1 primarily binds and cross-links lipid-poor ApoA-I, demonstrating minimal interaction with smaller HDL3 and no interaction with larger HDL2 subtypes (13, 14). Consequently, the interaction between ABCA1 and the predominant HDL subtypes present in plasma is restricted. ABCA1 is believed to have a central role in the initiation of cholesterol efflux from macrophages and other cells to lipid-poor apolipoproteins, whereas SR-BI primarily aids in the removal of cholesterol and cholesterol esters from large HDL particles. Furthermore, mature HDL particles can also acquire cholesterol through aqueous diffusion (AD). AD involves a straightforward diffusion mechanism in which cholesterol esters disengage from the endoplasmic reticulum or HDL particles, and are transported back to the cell membrane through cholesterol transporters such as ABCA1 and ABCG1. Subsequently, these cholesterol esters are released into the extracellular milieu, gathered, and conveyed to the liver by HDL particles for the synthesis of bile acids (4).

### 2.2 The physiological role of macrophage cholesterol efflux

Throughout the process of monocyte differentiation into macrophages, notable alterations in lipid metabolism frequently take place. Due to the lack of regulation by cellular cholesterol levels, SR can lead to uncontrolled uptake of modified LDL particles, surpassing the cell’s ability to store and release lipids. This results in the accumulation of cholesterol within macrophages and the development of foam cells (4). An overabundance of cholesterol uptake disrupts cellular equilibrium, leading to the activation of inflammatory signaling pathways that control the generation of reactive oxygen species (ROS), oxidative cytokines, and chemokines, exacerbating the situation (15). The formation of foam cells serves as an initial indicator of the development of atherosclerotic plaques. By facilitating cholesterol efflux, macrophages can uphold intracellular homeostasis and impede the onset and advancement of this process (16). Furthermore, MCE has been shown to effectively mitigate intracellular cholesterol accumulation, attenuate the activation of inflammatory pathways, suppress the release of inflammatory cytokines, and modulate macrophage polarization, thereby exerting a significant influence on both local and systemic inflammatory processes (15). Additionally, the regulation of MCE has the potential to enhance systemic insulin sensitivity, thereby aiding in the prevention and management of diabetes and its associated complications (17).

## 3 The relationship between macrophage cholesterol efflux and diabetic kidney disease

### 3.1 The impact of a high-glucose environment on macrophage cholesterol efflux

**Figure 1:**
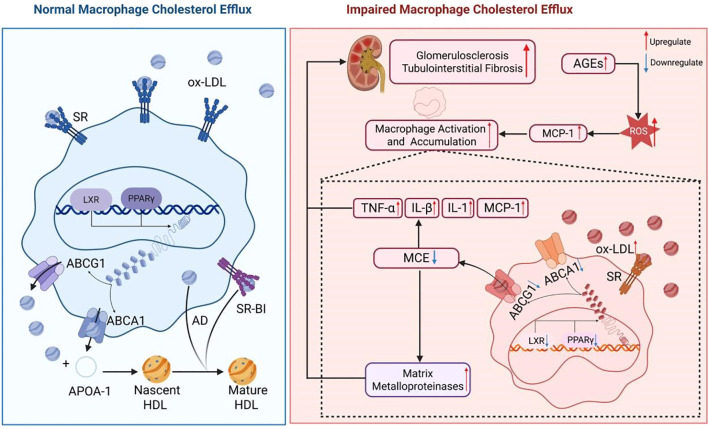
Effects of impaired macrophage cholesterol efflux in diabetic kidney disease. In the presence of elevated glucose levels, the body initiates an inflammatory cascade by releasing pro-inflammatory cytokines including TNFα, IL-8, IL-1β, and MCP-1. This process results in a notable influx of macrophages into renal tissue, leading to the injury of glomerular endothelial cells and mesangial cells. Furthermore, the released inflammatory mediators promote LDL oxidation by interacting with Toll-like receptors on the macrophage surface. The activation of IRF3, NF-κB, and AP1 leads to the release of inflammatory factors from macrophages and the generation of ROS, thereby exacerbating renal inflammation. Furthermore, these inflammatory factors suppress the expression of nuclear transcription factors LXR and PPARγ, impacting the expression of ABCA1 and ABCG1 genes, ultimately resulting in impaired cholesterol efflux in macrophages.

An elevated glucose concentration in the renal blood vessels can stimulate inflammatory reactions and the secretion of cytokines and macrophage chemoattractant protein-1 (MCP-1), which attract macrophages. These macrophages utilize Toll-like receptors 2 and 4 to internalize saturated fatty acids, activating themselves and initiating inflammatory signaling cascades involving interferon regulatory factor 3 (IRF3), activator protein 1 (AP1), and nuclear factor κB (NF-κB). This ultimately results in the impairment of glomerular endothelial cells and mesangial cells (18, 19). The inflammatory mediators generated as a result can exacerbate LDL oxidation and suppress the transcription of LXR and PPARγ, consequently diminishing the levels of ABCA1 and ABCG1 expression, leading to impaired MCE. A study by Aécio Lopes de Araújo Lira et al. revealed that albumin derived from individuals with Type 2 Diabetes Mellitus and an estimated glomerular filtration rate (eGFR) below 60 mL/min/1.73 m² undergoes heightened carbamylation, impacting MCE facilitated by HDL2 and HDL3. This process may facilitate the accumulation of lipids in macrophages and disrupt the mechanism of reverse cholesterol transport (RCT). Prior research has demonstrated that albumin extracted from the serum of individuals with poorly controlled diabetes or from rats with induced uremia can hinder macrophage reverse cholesterol transport by decreasing the expression of ABCA1 and ABCG1 (20). Correspondingly, Joseph et al. observed a notable decrease in ABCA1 and ABCG1 levels in mesangial cells of mice with diabetic nephropathy, aligning with these results (21). Furthermore, the buildup of advanced glycation end products (AGEs) may stimulate the secretion of inflammatory cytokines such as interleukin-8 (IL-8). Xiaoer Tang et al. conducted experiments on cholesterol transport to show that IL-8 effectively suppresses ApoA-I-mediated, ABCA1-dependent MCE by upregulating miR-18322 expression (22). (Shown in **Figure 1**).

### 2.2 The impact of impaired macrophage cholesterol efflux on diabetic kidney disease

Renal biopsies of patients with DKD demonstrate that macrophages are the predominant infiltrating leukocytes in both the glomeruli and tubulointerstitium. The degree of macrophage infiltration is positively associated with the development of glomerulosclerosis, tubular atrophy, and interstitial fibrosis (23). Dysregulation of macrophages, characterized by increased cholesterol uptake and subsequent production of inflammatory mediators, can lead to further complications such as LDL oxidation, endothelial cell activation, and recruitment of monocytes. Ox-LDL has been shown to induce the secretion of tumor necrosis factor-α (TNF-α), interleukin-1β (IL-1β), and MCP-1, leading to increased macrophage infiltration in renal tissue and worsening kidney damage (24, 25). Additionally, the accumulation of foam cells, originating from macrophages, in the glomeruli and tubules plays a crucial role in the development of glomerulosclerosis and interstitial fibrosis. The matrix metalloproteinases released by foam cells have the ability to break down the extracellular matrix, disrupt the glomerular basement membrane, and damage interstitial structures, thereby hastening the progression of glomerulosclerosis and tubular fibrosis (26). Furthermore, compromised MCE leads to excessive production of ROS by macrophages, resulting in direct harm to glomerular and tubular cells and exacerbating renal damage (27). The accumulation of intracellular lipids due to impaired MCE also contributes to increased cell membrane stiffness, which impairs the macrophage’s ability to recognize and bind apoptotic cells. This disruption hinders the signaling of receptors like MERTK, suppresses the expression of genes related to phagocytosis, and diminishes phagocytic effectiveness, ultimately exacerbating kidney injury and inflammatory reactions (28). (Shown in **Figure 1**).

### 2.3 Association between impaired macrophage cholesterol efflux and clinical biomarkers in diabetic kidney disease

MCE represents a critical pathological mechanism in metabolic diseases such as DKD and shows strong correlations with several clinical biomarkers. In a cohort study involving 220 patients with diabetes and 70 healthy controls, Ahmet Karatas et al. reported that the monocyte/HDL ratio (MHR) was significantly higher in patients with DKD compared with those with normoalbuminuric diabetes and healthy individuals. Moreover, MHR was positively associated with the urinary albumin-to-creatinine ratio (UACR), suggesting its potential as a biomarker for monitoring proteinuria progression in DKD (29). Similarly, Barati F et al. demonstrated that exposure of the human monocyte cell line U937 to platelets and ox-LDL(80 µg/ml) markedly upregulated the expression of CD36, ABCA1, SR-B1, ACAT1, and LXRα in macrophages, implying that elevated platelet activation and ox-LDL accumulation may contribute to cholesterol imbalance and foam cell formation (30). Therefore, assessment of ox-LDL levels and platelet activity may provide valuable insight into the progression of DKD. Furthermore, cathepsins L and S have been found to promote LDL degradation while suppressing cholesterol efflux, exacerbating lipid accumulation and foam cell development. Elevated serum levels of these proteases may thus serve as indirect indicators of MCE impairment in DKD (31).

## 4 Potential therapeutic strategies to restore macrophage cholesterol efflux in diabetic kidney disease

### 4.1 Targeting lipid transport proteins regulation

Transcription factors LXR/RXR regulate genes that mediate MCE (APOE, ABCA1, and possibly ABCG1), transport (LPL, CETP, and several genes encoding ApoC isoforms), cholesterol conversion to bile acids (CYP7A), and metabolism and excretion into bile or intestinal lumen (ABCG5 and ABCG8) (7). LXR agonists have been shown to decrease lipid accumulation in macrophages that infiltrate renal tissue, thereby mitigating the activation of multiple signaling pathways, including JNK1, JNK2, and NF-κB, resulting in decreased levels of pro-inflammatory cytokines such as IL6 and TNFα (21). Furthermore, treatment of macrophages with LXR or RXR activators can enhance ABCA1 mRNA expression and facilitate cholesterol efflux to ApoA-I (7). For example, Tall et al. demonstrated that administration of LXR agonists to Apo E KO mice resulted in increased expression of ABCA1 and ABCG1 mRNA in lesions (7). Similarly, LXR-623, an LXRβ agonist, has been shown to produce similar effects (32). Currently, two synthetic LXR agonists (T0901317 and GW3965) have been identified as potential therapeutic agents for DKD, primarily through the upregulation of sterol regulatory element-binding transcription factor 1c (SREBP-1c) to stimulate lipogenesis, leading to excessive secretion of triglycerides into the systemic circulation. Nevertheless, the clinical application of these therapies is impeded by hepatotoxicity. In response to this challenge, researchers have engineered innovative sHDL nanoplatforms featuring a KT peptide surface and a hydrophobic core incorporating LXR agonists. These nanoplatforms have the ability to circumvent the glomerular filtration barrier, facilitate mesangial retention, and augment cholesterol efflux, thereby presenting a potentially efficacious therapeutic option for individuals with DKD (21).PPARγ, a crucial transcription factor in macrophage cholesterol metabolism, has the potential to enhance ABCA1 transcription and cholesterol efflux by upregulating LXRα and ABCA1 sequentially (33). Recent studies have demonstrated that mangiferin may stimulate MCE through the PPARγ-LXRα-ABCA1/G1 pathway (34). Nevertheless, despite the identification of a genuine PPARγ response element in the LXRα gene, recent studies have not been able to validate the induction of ABCA1 mRNA and cholesterol efflux in macrophages by PPARγ agonists like troglitazone or rosiglitazone (35–37). It is proposed that the limitation in ABCA1 expression in differentiated cells may be attributed to the availability of LXR/RXR ligands rather than the abundance of LXRs. Due to the limited responsiveness of PPARγ to endogenous fatty acids, it has been observed that oxidized derivatives of fatty acids, specifically those containing circulating ox-LDL, can effectively stimulate PPARγ activation and facilitate the efflux of cholesterol (5).What’s more,the primary role of microRNA miR-33a, situated within the intron of the transcription factor sterol regulatory element-binding protein 2 (SREBP-2), is to suppress the expression of ABCA1 (38). Research conducted by Jenika D. Marshall demonstrated that lipoprotein lipase (LPL) hinders the gene expression of ABCA1, ABCG1, and SR-BI via the Akt pathway after 18 hours, establishing LPL as a crucial mediator in the process through which it inhibits cholesterol efflux (39). Consequently, the inhibition of microRNA miR-33a and Akt has the potential to enhance cholesterol efflux in macrophages. Another approach to augment efflux capacity involves the utilization of cholesterol ester transfer protein (CETP) inhibitors, which elevate HDL levels by way of the ABCG1 transporter (5). Furthermore, research has demonstrated that resolvin T4 (RvT4) can stimulate MCE through the SR-BI-neutral cholesterol ester hydrolase (NCEH) pathway (40).

Targeting ABCA1 and ABCG1 to regulate cholesterol efflux in MCE pathways is a promising strategy.While reduced ABCA1 expression alone is not enough to induce DKD, experimental manipulation through genetic or pharmacological means to increase ABCA1 levels has shown promising results in mitigating kidney disease progression, indicating ABCA1 as a potential target for therapeutic intervention. Experimental investigations have demonstrated that Tetramethylpyrazine-Paeoniflorin (TP), sorbitol A, gypenoside monomer–gypenoside XVII (GP-17), curcumin, LCBP, and 17β-estradiol estrogen receptor A can enhance MCE and inhibit lipid accumulation through the upregulation of ABCA1 and ABCG1 expression (41–46). Qianxia Yin et al. demonstrated that photobiomodulation therapy (PBMT) enhances ABCA1 expression and facilitates cholesterol efflux in lipid-loaded primary peritoneal macrophages through the activation of the phosphatidylinositol 3-kinase/protein kinase C zeta/specific protein 1 signaling cascade (47). Conversely, Min Zhang and team identified that stabilizing ABCA1 through the inhibition of protein degradation can elevate ABCA1 levels and improve cholesterol efflux (48).

### 4.2 Anti-inflammatory strategies

Excessive cholesterol accumulation in macrophages situated in an inflammatory microenvironment triggers the activation of the Triggering Receptor Expressed on Myeloid Cells 2(TREM2) and AMP-activated protein kinase (AMPK) signaling pathways. This activation subsequently leads to the upregulation of LXR, which in turn facilitates the expression of downstream genes such as ABCA1 and ABCG1, thereby modulating lipid metabolism in macrophages (49, 50). When the lipid burden surpasses the macrophage’s intrinsic regulatory abilities, a transformation into foam cells occurs. The collective action of inflammasomes and ox-LDL intensifies macrophage oxidative stress and inflammatory reactions, consequently impeding MCE (15). Hence, the implementation of anti-inflammatory approaches is crucial in enhancing MCE. Jun Mei et al. discovered that TP has the ability to decrease the secretion of TNFα, IL-1β, and MCP-1 induced by ox-LDL, which is a significant finding in the context of inflammation alleviation (41). Additionally, Metformin, a widely prescribed medication for diabetes, has been shown to effectively slow down the progression of DKD by enhancing cholesterol efflux and lipid metabolism via the activation of the AMPK pathway (51).As emerging therapeutic agents for DKD, sodium-glucose cotransporter 2 (SGLT2) inhibitors induce glycosuria, thereby shifting the primary cellular energy substrate from glucose to free fatty acid (FFA) oxidation. This metabolic reprogramming helps decrease intracellular levels of toxic lipid intermediates in podocytes, mesangial cells, and proximal tubular cells, ultimately mitigating kidney injury caused by excessive cholesterol accumulation (52). Moreover, glucagon-like peptide-1 receptor agonists (GLP-1RAs) have been shown to suppress activation of the NF-κB inflammatory pathway and reduce the release of proinflammatory cytokines such as IL-6, TNF-α, and MCP-1, while promoting macrophage polarization toward the anti-inflammatory M2 phenotype rather than the proinflammatory M1 phenotype—thus exerting beneficial effects on macrophage cholesterol metabolism. Clinically used GLP-1RAs, including exenatide and liraglutide, have been demonstrated to lower IL-10 levels in the kidneys of diabetic mice, an effect further confirmed *in vitro* in human monocytes (53).

Macrophage polarization involves the differentiation of macrophages into distinct functional states in response to various signals within the microenvironment, resulting in the classification of M1 macrophages as pro-inflammatory and M2 macrophages as anti-inflammatory (54). In a pro-inflammatory setting, M1 macrophages have been shown to suppress the expression and activity of ABCA1 and ABCG1, thereby promoting intracellular cholesterol buildup (55). Hence, enhancing the M1/M2 conversion of macrophages may lead to improved cholesterol efflux function. For instance, the activation of the miR-182-5p/HDAC9 signaling pathway by GP-17 can facilitate the transition of macrophages to the M2 phenotype (43). In order to enhance both cellular cholesterol efflux and targeted drug delivery to macrophages, researchers have developed a ROS-responsive PF/TC-AT-d-rHDL, which effectively enhances cholesterol clearance in foam cells upon exposure to ROS, inhibits intracellular lipid deposition, and promotes M2 polarization of macrophages (56). Moreover, research conducted by Saba Soltani et al. has demonstrated that PON1 and PON2, both paraoxonases implicated in HDL-mediated cholesterol efflux, play a crucial role in safeguarding cholesterol-laden foam cells. Specifically, PON1 functions to diminish ox-LDL formation, while PON2 acts as a defense mechanism against oxidative stress. Consequently, enhancing the activity of PON1 and PON2 has the potential to mitigate cholesterol accumulation and facilitate efflux (5). Additionally, lipophagy, a specialized autophagic process responsible for degrading intracellular lipid droplets (LDs), liberates free cholesterol and fatty acids. Promoting cholesterol efflux can be achieved by enhancing lipophagy through the selective knockdown of genes located on lipid droplets, including SQSTM1/p62, NBR1, and OPTN (57).

### 4.3 Interventions Targeting the MCE Pathway and Their Potential for Prognostic Improvement

Emerging therapeutic agents for DKD, including liraglutide and canagliflozin, have been shown to activate AMPK and downstream MCE-related signaling pathways, highlighting the central role of MCE in metabolic regulation, inflammation resolution, and renal protection (58, 59). In the SUSTAIN-6 trial, treatment with the GLP-1 receptor agonist semaglutide significantly reduced the risk of new-onset or worsening nephropathy compared with placebo (HR = 0.64, 95% CI: 0.46–0.88; P < 0.01), suggesting that its renoprotective effects may be mediated through improved metabolic homeostasis and anti-inflammatory mechanisms (60). Likewise, findings from the CANVAS program demonstrated that the SGLT2 inhibitor canagliflozin slowed the decline in eGFR, reduced UACR levels, and lowered the incidence of composite renal outcomes, further supporting its protective role against renal function deterioration (61, 62). Collectively, these data indicate that modulation of MCE may represent a pivotal mechanism by which metabolic agents confer renal benefits. In the future, therapeutic strategies targeting MCE may drive a paradigm shift in DKD management—from conventional metabolic control toward integrated immunometabolic modulation—thereby offering new opportunities for precision treatment and long-term outcome improvement. However, it is worth noting that direct pharmacological modulators of MCE remain at the preclinical research stage, and further studies are warranted to translate these findings into clinical practice (63).

## 5 Conclusion

Recent research has elucidated the mechanism of MCE, highlighting the importance of lipid metabolism in macrophages, particularly the role of surface proteins ABCA1 and ABCG1 in transporting cholesterol bound to ApoA-I and HDL for recycling in the liver. A stable lipid metabolism in macrophages is essential for internal homeostasis and the inhibition of inflammatory mediator release. Recent studies have shown that modulating lipid transport proteins, crucial transcription factors in cholesterol metabolism, and anti-inflammatory approaches can effectively modulate macrophages to enhance cholesterol efflux. Despite advancements in comprehending MCE, there are still constraints.Most studies have focused on the link between MCE and atherosclerosis, whereas direct investigations of MCE in the context of DKD, both in experimental models and clinical settings, remain limited. Moreover, although certain novel agents can indirectly enhance MCE, no therapeutics specifically targeting MCE have yet been developed or approved for clinical use.
